# Cold Atmospheric Plasma Suppressed MM In Vivo Engraftment by Increasing ROS and Inhibiting the Notch Signaling Pathway

**DOI:** 10.3390/molecules27185832

**Published:** 2022-09-08

**Authors:** Miao Qi, Xinyi Zhao, Runze Fan, Xinying Zhang, Sansan Peng, Dehui Xu, Yanjie Yang

**Affiliations:** 1 State Key Laboratory of Electrical Insulation and Power Equipment, Centre for Plasma Biomedicine, Xi’an Jiaotong University, Xi’an 710049, China; 2 The School of Life Science and Technology, Xi’an Jiaotong University, Xi’an 710049, China; 3 Department of Cardiovascular Medicine, First Affiliated Hospital of the Medical School, Xi’an Jiaotong University, Xi’an 710049, China

**Keywords:** CAP, MM, ROS, Notch

## Abstract

Multiple myeloma (MM) is the second most common hematologic malignancy. MM stem cells (MMSCs) are thought to be the main causes of in vivo engraftment and eventual recurrence. As a notable new technology, cold atmospheric plasmas (CAPs) show a promising anti-tumor effect, due to their production of various ROS. In this study, we found that different types of plasma could inhibit MM’s ability to form cell colonies, suppress MM in vivo engraftment, and extend survival times. We demonstrated that NAC (a ROS scavenger) could block ROS increases and reverse the inhibition of MM’s cell-colony-formation ability, which was induced by the plasma treatment. By using a stem cell signaling array, we found that the Notch pathway was inhibited by the plasma treatment; this was further confirmed by conducting real-time PCRs of three MM cell lines. Together, these results constitute the first report of plasma treatment inhibiting MM in vivo engraftment and prolonging survival time by suppressing the Notch pathway via ROS regulation.

## 1. Introduction

Multiple myeloma (MM) is the second most common hematologic malignancy, accounting for approximately 1% of neoplastic diseases and 10% of hematologic cancers [1,2].

MM is an incurable, biologically heterogeneous plasma cell disease, characterized by the accumulation of clonal malignant plasma cells in the bone marrow, which leads to the overproduction of non-functional intact immunoglobulins or immunoglobulin chains [3]. MM often causes end-organ damage, including anemia, hypercalcemia, renal failure, and bone lesions [4]. The main MM treatment methods include radiotherapy, chemotherapy, autologous stem cell transplantation, proteasome inhibitors, and immunomodulators [5,6,7,8,9]. Although most MM patients achieve a certain degree of remission, the disease is still difficult to cure and the majority of patients relapse [10]. Cancer stem cells (CSCs), a rare population of tumor cells, have self-renewing, multipotent, and highly tumorigenic abilities, and play an important role in promoting tumor initiation, recurrence, and metastasis [11,12,13]. MM stem cells (MMSCs) are considered to be the main cause of drug resistance and eventual disease recurrence [14]. Therapeutic responses to MM need to be further explored.

Hypoxia is a common phenomenon in tumor tissue; a growing body of evidence indicates that a hypoxic environment is considered a niche for the CSCs of tumor tissues, and that it maintains reactive oxygen species (ROS) at low concentrations in CSCs [15]. In general, CSCs have a lower rate of metabolic activity and thus produce fewer ROS; as such, they are highly sensitive to increased ROS levels. CSCs in several tumors contain lower ROS levels and enhanced ROS defenses, due to the increased expression of free radical scavenging systems; the regulation of low ROS levels in CSCs may be a potential approach for the treatment of local and systemic tumors [16]. Additionally, many signaling pathways involved in the growth, proliferation, self-renewal, and differentiation of normal stem cells are abnormally activated or inhibited in tumorigenesis and in cancer initiation and development; these include the Wnt, Notch, NF-κB, and Hedgehog pathways, which regulate CSCs’ initial development, and which are all active in a wide range of human cancers, including MM [17]. Moreover, the Notch signaling pathway is deregulated in MM, and preclinical data suggest that this is related to the progression of MM [18].

In recent years, cold atmospheric pressure plasmas (CAPs) have developed rapidly and attracted much attention due to their potential applications, in particular their promise as a novel cancer treatment [19,20]. CAPs are of particular interest due to their ability to selectively kill cancer cells without damaging normal cells in vitro and in vivo [21]. CAPs produce various physicochemical factors, including electrons, ions, photons, a transient electric field, and a large amount of reactive oxygen and nitrogen species (ROS/RNS), which cause cancer cell death. It is worth noting that RONS are major biomedical effectors of CAP cancer treatments [22]. Several studies have demonstrated that CSCs overloaded with an abundance of RONS generated by CAPs may overcome this protective shield, which uses ROS-quenching enzymes to alleviate their toxic effects [23], suggesting that CAPs are a potential means of killing MMSCs and preventing MM recurrence.

In this study, we set out to prove that CAPs inhibit the cell-colony-formation abilities and in vivo engraftment of MM, using colony-forming cell (CFC) numbers and mouse survival time. In addition, we demonstrated the relationship between MM-cell-colony-formation ability and ROS produced by CAPs. We found that Ar plasma has good anti-MM-cell-colony-formation ability. We detected the physicochemical properties of Ar plasma by discharge imaging: these properties include the waveforms of the voltage and current measurement, the optical emission spectrum (OES), the concentrations of H_2_O_2_ and NO_2_^−^, the pH value, and the ORP. Moreover, the cancer stem cell PCR array, the mRNA level, and the Notch inhibitor (DAPT) indicated that CAPs downregulate the Notch signaling pathway and inhibit MM-cell-colony-formation abilities.

## 2. Results

### 2.1. Plasma-Inhibited MM-Cell-Colony-Formation Ability In Vitro and Engraftment In Vivo

**Figure 1:**
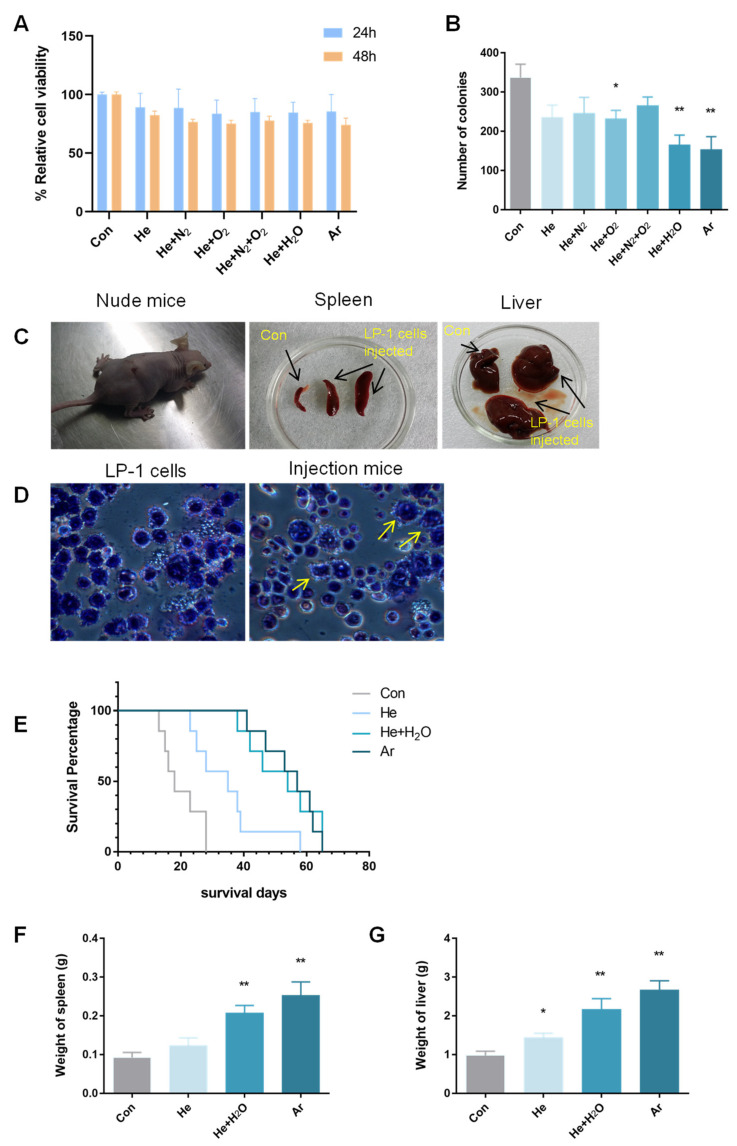
Plasma inhibited MM cell colony formation in vitro and engraftment in vivo. (**A**) Cell viability at 24 h and 48 h after pure He, He + O_2_ (0.5%), He + N_2_ (0.5%), He + O_2_ (0.5%) + N_2_ (0.5%), He + H_2_O (1%), and pure Ar plasma treatment. (**B**) CFC assays were performed after various plasma treatments. Cells were plated at a concentration of 10,000 per mL (LP-1). The number of colonies was counted and presented as the mean ± SD (*n* = 3). (**C**) Construction of the MM mouse xenograft model, and images of the spleens and livers of control mice and LP-1 mice. (**D**) Giemsa Staining with LP-1 cells and the bone marrow of the LP-1 mice, arrows represent LP-1 cells in mice bone marrow. (**E**) Kaplan–Meier survival analysis (*n* = 7). The *x*-axis represents the number of survival days from injection to the development of hind leg paralysis, and the *y*-axis represents the survival percentage. Then, (**F**) spleens and (**G**) livers were collected and weighed. Data represent the mean ± SD; * *p* < 0.05; ** *p* < 0.01 (Student’s *t* test).

Previous studies have shown that plasma can efficiently induce MM cell apoptosis [24]. To explore the role of plasma in the MM-cell-colony-formation ability in vitro, we first analyzed plasma that slightly inhibited the effects of MM cell growth, in order to rule out excessive cell death. We used pure He, He + O_2_ (0.5%), He + N_2_ (0.5%), He + O_2_ (0.5%) + N_2_ (0.5%), He + H_2_O (1%), and pure Ar gases for experiments. LP-1 cells were used as a model for plasma treatment. Following treatment with the various plasmas for 20 s, cell viability could be detected after 24 h and 48 h by a Cell-Titer-Glo^®^ luminescent cell viability assay. As shown in Figure 1A, 24 h and 48 h after plasma treatment, the growth of LP-1 cells was not significantly inhibited (*p* > 0.05). Many studies have demonstrated that CSCs have sphere formation abilities and tumorigenicity in vivo; the CFC assay and in vivo tumorigenesis assay are the gold-standard assays for the assessment of the self-renewal potential of cancer stem cells in vitro [25]. In Figure 1B, after the various plasma treatments for 20 s, LP-1 cells were mixed with the MethoCult medium, and the number of colonies counted for the last 14–21 days. As was the case with cell viability, Ar plasma had the best treatment effect, and the average number of colonies was 153, suggesting that plasma inhibited MM cell colony formation. MM cells can invade and damage the functions of other tissues and organs, such as the spleen and liver [26]. In a syngeneic murine model of MM that shared many characteristics with the human disease, MM cells were found in the spleen, bone marrow, and liver [27]. We established that, in the MM xenograft mouse model (Figure 1C,D), the spleens and livers of the mice were enlarged, and tumor cells were found in the mice. In Figure 1E, following treatment with the various plasmas for 20 s, the cells were transplanted into the tail veins of mice, and we monitored the survival curve of these mice. The results showed that the mice in the Ar plasma group had the longest average survival time, while the He plasma group had the shortest survival time. Perhaps because the mice in the Ar plasma group survived the longest, the spleens and livers of these mice were the heaviest (Figure 1F,G).

### 2.2. Plasma Suppressed MM-Cell-Colony-Formation Abilities by Increasing ROS

**Figure 2:**
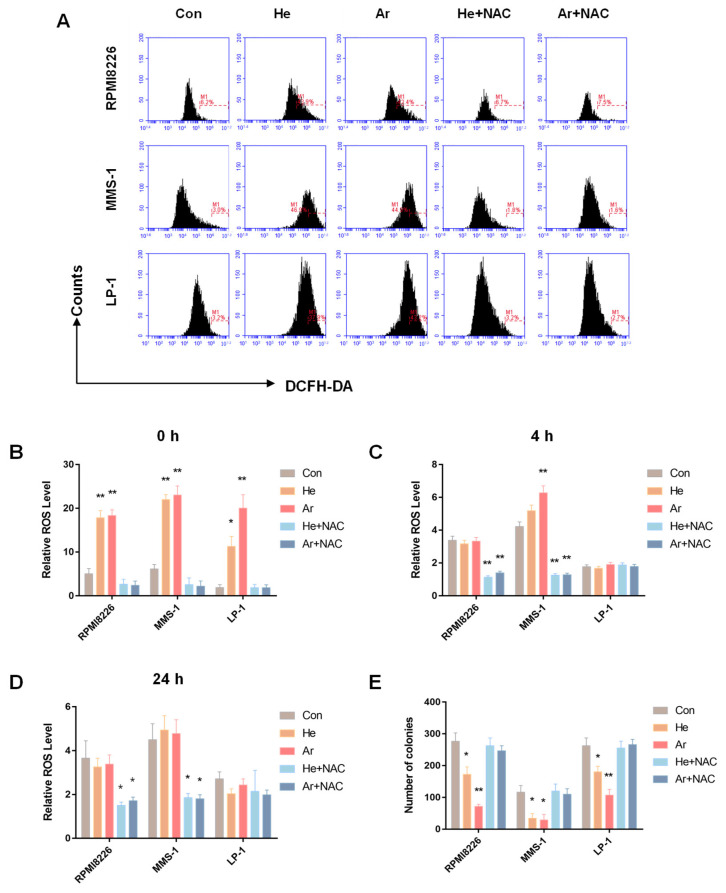
Plasma suppressed MM-cell-colony-formation abilities by increasing ROS. (**A**) RPMI8226, MMS-1, and LP-1 cells were pretreated with the ROS scavenger NAC (200 μM), and then treated with He and Ar plasma for 20 s, followed by incubation with 10 μM DCFH-DA for 20 min. DCF fluorescence was examined and quantified, ROS levels were measured at (**B**) 0 h, (**C**) 4 h, and (**D**) 24 h after plasma treatment. (**E**) Cells had been treated with NAC and plasma were then plated in CFC assays to assess clonogenic ability. Data represent the mean ± SD; * *p* < 0.05; ** *p* < 0.01 (Student’s *t* test).

It is well known that plasma discharge can produce a high number of ROS. To verify the role of ROS in the regulation of MM cell colony formation during plasma treatment, we utilized flow cytometry for 20 s to test the base ROS levels of the various plasma treatments. In Figure 2A, RPMI8226, MMS-1, and LP-1 cells were treated by He and Ar plasma; three cells’ cellular ROS levels were upregulated after the plasma treatment, and decreased to control levels after the use of the ROS scavenger NAC. In addition, as the time after the plasma treatment increased, the level of ROS in the cells decreased. After about 4 h, the ROS numbers recovered more or less to the level of the control group, and after 24 h, the number of ROS was the same as that of the control group (Figure 2B–D). To explore the relationship between ROS and MM-cell-colony-formation abilities, we treated three kinds of cells with He and Ar plasma for 20 s, then mixed them with the MethoCult medium for a CFC assay. The results showed that the number of cell colonies decreased after plasma treatment, but that the number of cell clones was recovered after using NAC (Figure 2E).

### 2.3. Physicochemical Properties of Ar Plasma

**Figure 3:**
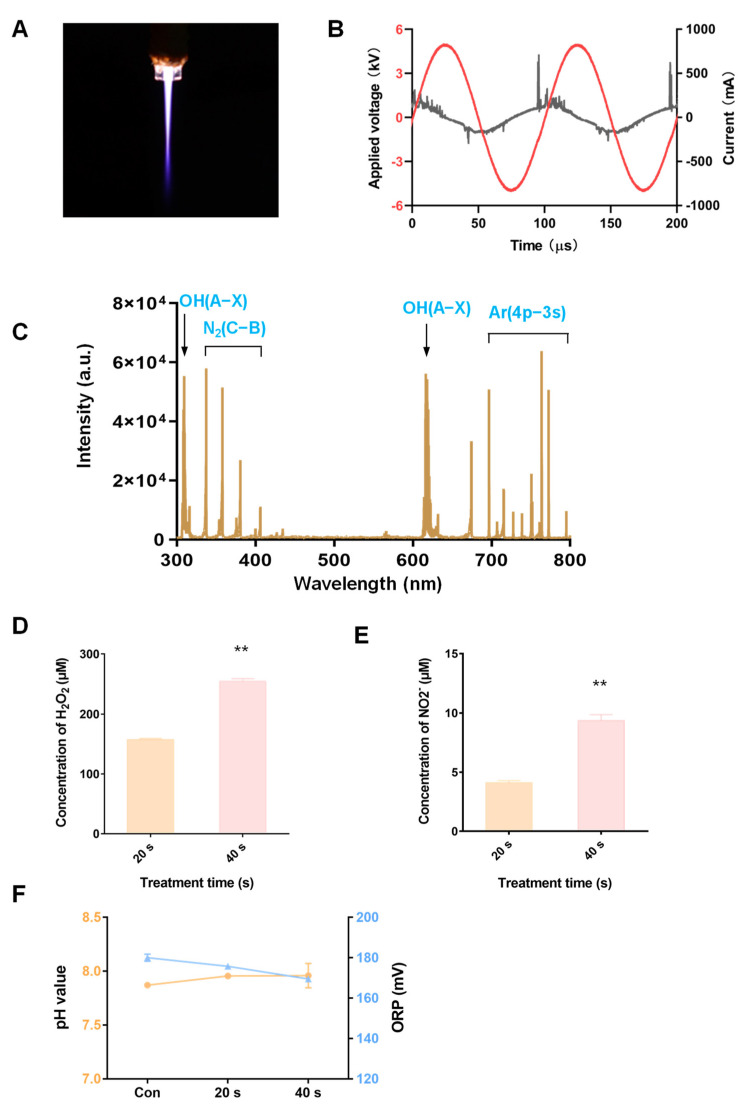
Physicochemical properties of Ar plasma. (**A**) The discharge image, (**B**) the waveforms of the voltage and current, and (**C**) the optical emission spectrum (OES) of Ar plasma. When Ar plasma treated the cell culture medium for 20 s and 40 s, the concentrations of (**D**) H_2_O_2_ and (**E**) NO_2_^−^, and (**F**) the pH value and ORP were measured. Data represent the mean ± SD; ** *p* < 0.01 (Student’s *t* test).

Ar was more effective in inhibiting MM-cell-colony-formation abilities, as shown in Figure 1 and Figure 2. Next, we elucidated the physicochemical properties of Ar plasma. The image of the Ar plasma jet discharged is shown in Figure 3A; the current and voltage waveforms are shown in Figure 3B. To determine the composition of the main products produced by the Ar plasma, these products were analyzed by OES from 200 to 800 nm. The spectrum was mainly composed of OH(A-X) at 309 and 616 nm, the second positive bands of N_2_(C-B) at 337 nm, and Ar(4p→3s) at 696 nm (Figure 3C) [28,29]; the OES of other plasma gases are shown in Supplementary Figure S1. The plasma contained a wide variety of reactive species, such as H_2_O_2_ and NO_2_^−^, which have good anti-cancer effects [30]. In Figure 3D,E, which show the Ar-plasma-treated cell medium at 20 s and 40 s, the concentration of H_2_O_2_ is shown to increase from 157.06 µM to 254.43 µM, while NO_2_^−^ increased from 4.09 µM to 9.37 µM. In Figure 3F, the pH value increased slightly, but the oxidation-reduction potential (ORP) of the medium was decreased after plasma treatment, and the ORP value of the PAM decreased from 180 to 169.5 mV. The ORP is generally employed to indicate global ROS levels in the plasma-activated medium, probably because the medium constitutes a complex solution environment which contains organic components, inorganic particles, and a buffer system.

### 2.4. Plasma Suppressed MM-Cell-Colony-Formation Abilities by Inhibiting the Notch Pathway

**Figure 4:**
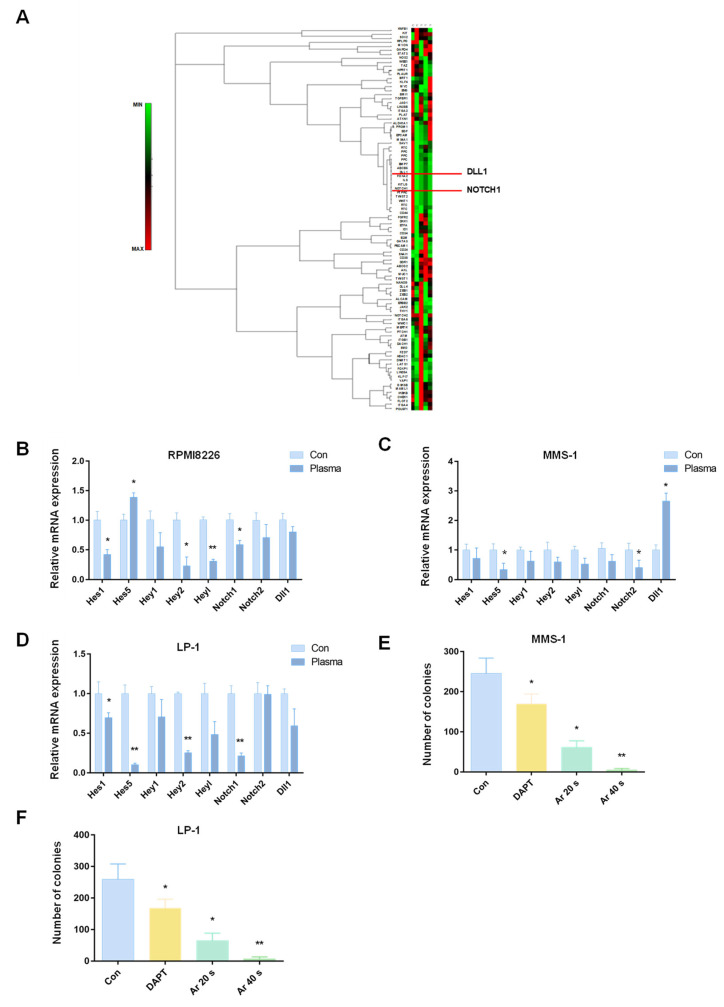
Plasma suppressed MM cell colony formation by inhibiting the Notch pathway. (**A**) Heatmap of cancer stem cell PCR arrays of LP-1 cells after Ar plasma treatment. Real-time PCR was performed for Notch-pathway-related genes (Hes1, Hes5, Hey1, Hey2, Heyl, Notch1, Notch2, and Dll1) after 24 h of Ar plasma treatment in (**B**) RPMI8226, (**C**) MMS-1, and (**D**) LP-1 cells. (**E**) MMS-1 and (**F**) LP-1 cells were treated with 10 μM DAPT for four days, and treated with Ar plasma for 20 s and 40 s, then plated in the CFC assay to assess clonogenic ability. Data represent the mean ± SD; * *p* < 0.05; ** *p* < 0.01 (Student’s *t* test).

To further characterize the mechanism by which plasma suppresses cell-colony-formation abilities, we examined the expression of stem cell signaling pathways in LP-1 cells using a cancer stem cell PCR array, as shown in Figure 4A. After Ar plasma treatment, many stem cell signaling pathways had changed; notably, the Dll1/Notch pathway was enriched. Notch signaling is a highly conserved and important pathway which is involved in many aspects of cancer biology, including CSC phenotypes, angiogenesis, metastasis, and tumor immune evasion [31]. To determine whether Notch signaling pathways play a major role in Ar plasma’s suppression of MM-cell-colony-formation abilities, we examined the mRNA levels of MM cells. As shown in Figure 4B–D, the mRNA levels of the Notch signaling pathway genes Hes1, Hes5, Hey1, Hey2, Heyl, Notch1, Notch2, and Dll1 were most significantly decreased after Ar plasma treatment. Finally, based on the fact that plasma inhibits MM cell growth, and that plasma suppresses the Notch signaling pathway, we used Notch inhibitor DAPT and Ar plasma to treat MMS-1 and LP-1 cells, as shown in Figure 4E,F. When we blocked the Notch pathway using DAPT and treatment with Ar plasma, the colony numbers were decreased.

## 3. Discussion

Increased ROS production is becoming a well-known hallmark of various cancer cells, compared to their normal counterparts, leading to genetic instability and tumorigenesis [32]. Meanwhile, the antioxidant capacity of tumor cells scavenges excess ROS while maintaining tumor-promoting ROS levels, making tumors resistant to apoptosis [33]. Increased ROS levels regulate many cell signaling pathways, such as mitogen-activated-protein kinase (MAPK)/extracellular-regulated kinase 1/2 (ERK1/2), phosphoinositide-3-kinase (PI3K)/Akt, protein kinase D (PKD), PTEN (phosphatase and tensin homolog deleted on chromosome ten), nuclear factor−κB (NF-κB), and Nrf2 (Nuclear Factor erythroid 2-Related Factor 2) [34]. However, ROS concentration at critical cytotoxic levels in cells is known to activate anti-cancer signaling pathways, and induce cell cycle arrest, apoptosis, and senescence [35,36,37].

ROS are short-lived species, which include superoxide (O^•^_2_^−^) and hydroxyl (OH^•^); they can be quickly transformed into more stable, freely diffusible non-free radicals, such as hydrogen peroxide (H_2_O_2_) and hypochlorous acid [38,39]. Many studies have indicated that a variety of RONS-reactive species, such as H_2_O_2_, NO_2_^−^, NO_3_^−^, NO, O_3_, ^1^O_2_, OH^•^, O_2_^−^, and ONOOH/ONOO^−^ are generated after plasma reacts with cancerous cells or tissues [40,41]. ROS and RNS are major effectors of the anti-cancer activity of plasma. As shown in Figure 2, RPMI8226, MMS-1, and LP-1 cells were treated using He and Ar plasma; the cellular ROS levels of three cells were upregulated after plasma treatment, and then decreased to control levels after the use of the ROS scavenger NAC. The ROS level was time-dependent, such that the ROS level was the same as that of the control group 24 h after the plasma treatment. This indicates that plasma could suppress MM cell growth by increasing ROS levels. Our previous study showed that plasma could efficiently induce MM cell apoptosis through the activation of CD95 and downstream caspase cascades [24]. In addition, plasma can promote MM differentiation by upregulating Blimp-1 and XBP-1 expression and decreasing MMP-2 and MMP-9 secretion to suppress the migration of MM cells. Plasma can also increase bortezomib sensitivity and induce myeloma cell apoptosis [42]. As we know, CSCs have sphere formation abilities and tumorigenicity in vivo. However, the use of plasma to inhibit MM-cell-colony-formation abilities in vitro and engraftment in vivo has rarely been reported. Ar plasma not only inhibited MM’s ability to form cell colonies, but also showed therapeutic anti-melanoma efficacy in vitro and in vivo [43].

Luo et al. stated that modulation of the redox signaling pathway could induce equilibrium in breast cancer stem cells [44]. Choi et al. verified that gastric CSCs increased oxidative phosphorylation (OXPHOS) and maintained low ROS levels; targeted ROS homeostasis in CSCs may be a useful strategy for targeting drug-resistant tumor cells [45]. Hou et al. eliminated stem-like cancer cell side-populations using auranofin to increase ROS levels and inhibit glycolysis in non-small lung cancer cells [46]. These studies demonstrate that increased ROS levels in CSCs may be utilized in the treatment of cancer and the prevention of tumor invasion, drug resistance, and recurrence after treatment. As shown in Figure 1B, when LP-1 cells were treated with plasma, colony numbers were decreased, suggesting that plasma can suppress MM-cell-colony-formation abilities. Moreover, plasma is a potential means of killing other CSCs; for example, Li et al. used air-based CAP and a plasma-activated medium (PAM) to selectively induce cell death in Hep3B and Huh7 cells with CSC characteristics, but not in the normal liver cell line [47].

The Notch signaling pathway is one of the most active pathways in many cancers. It is a highly conserved pathway that is related to many functions of cancer, including the CSC phenotype, angiogenesis, metastasis, and tumor immune evasion [48]. Additionally, the Notch pathway plays a key role in the link between angiogenesis and the self-renewal of CSCs. As shown in Figure 4, a cancer stem cell PCR array was used to determine that the Dll/Notch pathway was the signaling pathway most significantly altered after the Ar plasma treatment. Through real-time PCR, it was also concluded that the Notch signaling pathway was significantly changed after the plasma treatment of MM cells. Moreover, the number of cell clones decreased after Notch inhibition. With regards to MM specifically, Sabol et al. proved that Notch inhibitors reduce multiple myeloma growth, and reduce bone destruction caused by cancer [49]. In addition, Colombo et al. found that MM patients show gain-of-function mutations in Notch pathway members; moreover, in MM cells, Notch signaling is aberrantly activated [50]. Furthermore, Paul found that Nrf2 directly regulates Notch for stem cell self-renewal, and that the whole ROS-Nrf2-Notch pathway is key for cellular homeostasis [51]. To summarize, in this study, plasma was found to increase ROS, and destroy the dynamic regulation of ROS and the Notch signaling pathway in MM.

## 4. Materials and Methods

### 4.1. Plasma Jet Device

The schematic of the plasma jet used in this research was described in our previous study [52]. The atmospheric pressure plasma jet (APPJ) device adopts a coaxial configuration, using a stainless-steel tube as a high-voltage electrode and a copper ring wrapped around a gas-guiding quartz tube as the ground electrode. The vertical distance between the two electrodes was 5 mm. The funnel-shaped quartz tube (6 mm outer diameter) had a length of 7 cm; the inner diameter of the nozzle wall was 4 mm (with a length of 5 cm), while that of the other part was 6 mm (with a length of 2 cm). The power supply was a sinusoidal AC with a frequency of 10 kHz and a peak-to-peak voltage of 10 kV. The working gases He (purity = 99.999%), He + O_2_ (0.5%), He + N_2_ (0.5%), He + O_2_ (0.5%) + N_2_ (0.5%), He + H_2_O (1%), and pure Ar (purity = 99.999%) were supplied, and the total gas flow rate was controlled to be 2 SLM (standard liters per minute). We measured the voltage and current of the AC boost system using a high voltage probe (Tektronix, P6015A, OR, USA) and a current probe (Tektronix, P6021, OR, USA), and we observed it using an oscilloscope (Agilent, DSO-X 2014A, CA, USA). The discharge image was taken using a digital camera (Nikon D7000).

### 4.2. Cell Culture and Treatment and the Cell Viability Assay

RPMI8226, MMS-1, and LP-1 cells were kindly donated by Doctor Hu from the Department of Molecules and Genetics, at the medical school of Xi’an Jiaotong University. Cells were cultured in Roswell Park Memorial Institute (RPMI) 1640 (Corning, Jiangsu, China), and supplemented with 10% FBS (Gibco, NY, USA) and 1% penicillin–streptomycin (100×) (MedChemExpress, NJ, USA) at 37 °C in a 5% CO_2_ incubator. In total, 0.5 million cells were seeded in 12-well plates, supplemented with a complete medium, and treated with plasma for 20 s. Then, 24 and 48 h after plasma treatment, cell viability was determined using a Cell-Titer-Glo^®^ luminescent cell viability assay kit (Promega, WI, USA), following the manufacturer’s instructions. Independent experiments were repeated in triplicate.

### 4.3. Colony Forming Cell (CFC) Assay

Cells were treated with plasma (20 s) or with N-acetyl-L-cysteine (NAC) (Sigma, MO, USA), which was added prior to plasma treatment to guarantee its effectiveness at concentrations of 20 μM for NAC or 10 μM for DAPT (Sigma). After four days, cells were collected and suspended in Iscove’s MDM medium. MM cells (10,000/mL) were plated in a MethoCult medium (StemCell Technologies, BC, Canada ) and, after an incubation period of 14–21 days, the colonies were counted.

### 4.4. Animal Study

BALB/c nude mice (female) aged 6–8 weeks old were purchased from the Medical Animal Center of Xi’an Jiaotong University. The mice were allowed to adapt for one week prior to the experiment. Then, the mice were randomly divided into four groups (control, He, He + H_2_O, and Ar), with seven mice per group. LP-1 cells (0.5 × 10^6^) that had been pretreated with plasma (20 s) were injected intravenously into the nude mice. The mice were sacrificed when they developed hind leg paralysis. Survival days from injection were recorded. The experimental protocols were approved by the Hospital Research Ethics Committee of Xi’an Jiaotong University.

### 4.5. Giemsa Staining Detect

The Mix Giemsa Staining Solution (Beyotime, Shanghai, China) with mouse bone marrow was used, and a microscope (BX53, Olympus, Tokyo, Japan) was employed to capture the image. Mouse bone marrow cell isolation was performed as previously described [53].

### 4.6. Detection of ROS Levels

The intracellular ROS level was detected using the fluorescent probe DCFH-DA, according to the manufacturer’s instruction (Beyotime, China). The cells were incubated at 37 °C for 20 min and washed 3 times with a basic medium, then analyzed using flow cytometry (Accuri C6, BD Biosciences, CA, USA).

### 4.7. Real-Time PCR Assay

A real-time PCR analysis was performed as previously described [54]. The primers are listed in Table 1.

### 4.8. Cancer Stem Cell PCR Array

Total RNA was extracted from Ar-plasma-treated LP-1 cells and was analyzed using a human cancer stem cell PCR array from Thermo Fisher, according to the manufacturer’s instructions.

### 4.9. Measurement of Physicochemical Properties

The optical emission spectrum (OES) emitted by the reactive species in the Ar plasma plume was detected by an Andor SR-750i grating monochromator (grating grooving 1200 lines mm^−1^). The concentration of H_2_O_2_ was measured using the Hydrogen Peroxide Assay kit (Beyotime, S0038) and the concentration of NO_2_^−^ was measured using a Griess Reagent Kit (Beyotime, S0021). A microplate reader (Varioskan Flash Reader; Thermo Fisher Scientific, MA, USA) was used to detect the quantitative concentrations.

### 4.10. Statistical Analysis

All the data are presented as the means ± SDs. To determine the significant differences between the tested groups, Student’s t test was used. Data from the study were considered statistically significantly different at * *p* < 0.05 and ** *p* < 0.01. All the experiments were performed at least three times.

## Figures and Tables

**Table 1:** Primers of real-time PCR.

Gene	Forward Sequence	Reverse Sequence
Hes1	AGGCTGGAGAGGCGGCTAAG	TGGAAGGTGACACTGCGTTGG
Hes5	CCGGTGGTGGAGAAGATGCG	GCGACGAAGGCTTTGCTGTG
Hey1	GGATCACCTGAAAATGCTGCATAC	CCGAAATCCCAAACTCCGATAG
Hey2	GTGCGGCTTGTGTCTCATCTC	CTGCTGCTGCTGCGTTTG
HeyL	AGCCAGGAAGAAACGCAGAGG	GCTGTTGAGGTGGGAGAGAAGG
Notch1	GCCGCCTTTGTGCTTCTGTTC	GTCCTCCTCTTCCTCGCTGTTG
Notch2	CTCCTTGCTGTTGCTGTTGTCATC	CACATCCACCTCCTGCTCTGC
Dll1	GCCTGCCTGGATGTGATGAG	CTGGATAGCGGATACACTCGT
ActinB	GCCTCGCTGTCCACCTTCC	TGCTGTCACCTTCACCGTTCC

## Data Availability

Not applicable.

## Supplementary Materials

The following supporting information can be downloaded at: https://www.mdpi.com/article/10.3390/molecules27185832/s1, Figure S1: The optical emission spectrum of (A)He, He + O_2_ (0.5%), He + N_2_ (0.5%), He + O_2_ (0.5%) + N_2_ (0.5%), He + H_2_O (1%) plasmas.
