# Supplementary material for: Cold Atmospheric Plasma Suppressed MM In Vivo Engraftment by Increasing ROS and Inhibiting the Notch Signaling Pathway
Source: Molecules. 2022 Sep 8;27(18):5832. doi: 10.3390/molecules27185832 (PMC9501839; doi:10.3390/molecules27185832)
Supplement: Supplementary file 1 [file molecules-27-05832-s001.zip › molecules-1859366-supplementary.pdf]

Supplementary Figure S1

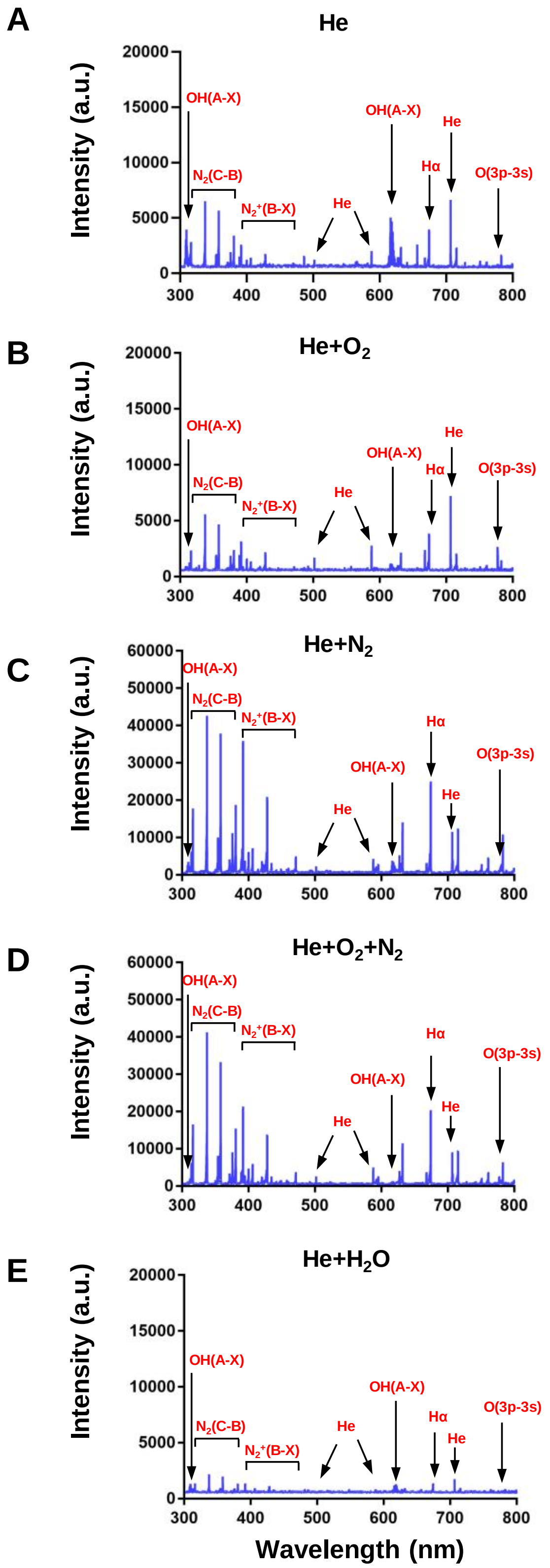

Supplementary Figure S1. The optical emission spectrum of (A) He, (B) He + O_2 (0.5%), (C) He + N_2 (0.5%), (D) He + O_2 (0.5%) + N_2 (0.5%) and (E) He + H_2O (1%) plasma.
